# Pulse Pressure Magnifies the Effect of *COMT* Val^158^Met on 15 Years Episodic Memory Trajectories

**DOI:** 10.3389/fnagi.2016.00034

**Published:** 2016-03-02

**Authors:** Ninni Persson, Catharina Lavebratt, Anna Sundström, Håkan Fischer

**Affiliations:** ^1^Department of Psychology, Stockholm UniversityStockholm, Sweden; ^2^The Center for Molecular Medicine, Karolinska University HospitalStockholm, Sweden; ^3^Department of Molecular Medicine and Surgery, Karolinska InstitutetStockholm, Sweden; ^4^Department of Psychology, Umeå UniversityUmeå, Sweden; ^5^Centre for Demographic and Ageing Research, Umeå UniversityUmeå, Sweden

**Keywords:** cognition, aging, *COMT*, hypertension, longitudinal, single nucleotide polymorphism, SNP

## Abstract

We investigated whether a physiological marker of cardiovascular health, pulse pressure (PP), and age magnified the effect of the functional *COMT* Val^158^Met (rs4680) polymorphism on 15-years cognitive trajectories [episodic memory (EM), visuospatial ability, and semantic memory] using data from 1585 non-demented adults from the Betula study. A multiple-group latent growth curve model was specified to gauge individual differences in change, and average trends therein. The allelic variants showed negligible differences across the cognitive markers in average trends. The older portion of the sample selectively age-magnified the effects of Val^158^Met on EM changes, resulting in greater decline in Val compared to homozygote Met carriers. This effect was attenuated by statistical control for PP. Further, PP moderated the effects of *COMT* on 15-years EM trajectories, resulting in greater decline in Val carriers, even after accounting for the confounding effects of sex, education, cardiovascular diseases (diabetes, stroke, and hypertension), and chronological age, controlled for practice gains. The effect was still present after excluding individuals with a history of cardiovascular diseases. The effects of cognitive change were not moderated by any other covariates. This report underscores the importance of addressing synergistic effects in normal cognitive aging, as the addition thereof may place healthy individuals at greater risk for memory decline.

## Introduction

Aging is characterized by a combination of stability, growth, and decline of cognitive abilities across the adult life span (de Frias et al., 2007a; Persson et al., 2016). More importantly, there is often a prominent pattern of differential rates of change across individuals in cognitive trajectories (Hultsch et al., 1992; Rabbitt et al., 2004; Ghisletta et al., 2012). Age-related changes also vary in degree across different cognitive domains, with working memory, episodic memory, processing speed, and visuospatial abilities exhibiting particular sensitivity to aging, whereas semantic memory shows age resistance until the end of life (Flicker et al., 1993; Small et al., 2011; Ghisletta et al., 2012).

Cardiovascular and genetic factors are important determinants of individual differences in cognitive decline. Genomic variability and cardiovascular-related health agents might underlay and interplay with cognitive aging. Findings from associative studies are limited and inconclusive, but some reports have found that cardiovascular risk × gene interactions can determine cognitive variability (de Frias et al., 2007b, 2014; Raz et al., 2008; Persson et al., 2013a).

With aging follows age-related loss of dopamine transmitters (Volkow et al., 1996; Bäckman et al., 2006), and genetic variants with the potential to influence dopamine levels are important for age-related cognitive changes. Catechol O-methyltransferase (*COMT*) degrades catecholamines (dopamine, norepinephrine, and epinephrine), and thereby affects catecholamine signaling. *COMT* modulates both nerve function and physiology due to broad distribution throughout the brain and in various peripheral tissues (Myöhänen et al., 2010). Val^158^Met (rs4680) is a single-nucleotide polymorphism (SNP) in the *COMT* gene (MIM 116790) that influences *COMT* enzymatic activity. The SNP implies an exchange of the amino acid valine (Val) to methionine (Met) at position 158 of the membrane-bound enzyme, and at position 108 of the soluble enzyme. Dopamine levels in the neocortex depend on *COMT* activity (Tunbridge et al., 2004; Myöhänen et al., 2010). The Val variant in the Val^158^Met polymorphism corresponds to higher *COMT* enzymatic activity (Chen et al., 2004; Tunbridge et al., 2004) in the prefrontal cortex, which presumably leads to lower synaptic dopamine levels by increased dopamine degradation (Lachman et al., 1996; Chen et al., 2004).

The functional *COMT* Val^158^Met polymorphism has attracted extensive attention in relation to cognitive function. The vast majority of studies are cross-sectional, and findings are to some degree inconsistent. A meta-analysis comprised of 12 studies and 1910 individuals (Barnett et al., 2007) reported small but significant effects on the association between Val^158^Met and a wide range of cognitive abilities. Advantages of homozygote Met carriers over Val carriers in tasks of episodic memory, spatial performance, and executive functions have been reported (Egan et al., 2001; de Frias et al., 2004, 2005; Barnett et al., 2007; Nagel et al., 2008; Raz et al., 2009). Greater risk for cognitive decline has been observed in both heterozygotes and homozygote carriers of the Val allele (Barnett et al., 2008; Wishart et al., 2011). In contrast, carriers of the Val allele have shown greater recall accuracy on tasks of episodic (O'Hara et al., 2006) and working memory (Wang et al., 2013). Studies have also shown little or no association between cognition and Val^158^Met (Barnett et al., 2008; Wardle et al., 2013). Variations in results may emerge from differences in study design and sampling procedures, but the presence of uncontrolled so-called third variables acting as moderators may also influence these effects. Important candidates for a moderator variable approach are cardiovascular risk factors (de Frias et al., 2007b, 2014; Raz et al., 2008; Persson et al., 2013a). The effects of the Val^158^Met polymorphism on cognition may also gain attention in older adults and individuals already at risk for cognitive decline (de Frias et al., 2005; Nagel et al., 2008; Josefsson et al., 2012; Papenberg et al., 2014).

Several studies have associated cognitive decline with a poorly controlled blood pressure (Waldstein, 2003; Waldstein et al., 2008; Persson et al., 2013b). *COMT* is a candidate gene for hypertension (Friese et al., 2011) since degradation of catecholamines plays a critical role in the regulation of vessel tone and blood pressure (Jordan et al., 2002; Guyenet, 2006). Experimental work show lower activity of membrane-bound *COMT* in the brain of spontaneously hypertensive rats (Masuda et al., 2006). Findings from epidemiological studies are somewhat inconclusive, linking the Val allele with hypertension and systolic blood pressure elevation (Hagen et al., 2007; Kamide et al., 2007). Counteracting has also carriers of the Met/Met allelic variant evidenced higher systemic blood pressure in alcohol dependents and female volunteers (Stewart et al., 2009; Yeh et al., 2010). Also, negative findings have been reported concerning pregnancy-induced hypertension (Sun et al., 2004).

The potential dual influence of *COMT* availability through dopaminergic regulatory pathways on cerebral dopamine levels and blood pressure regulation (Jose et al., 2003; Zeng et al., 2007) makes it interesting to examine potential interactive effects of blood pressure and allelic variants in the Val^158^Met polymorphism. Pulse pressure (PP) combines information about systolic and diastolic blood pressure, and is regulated by large arteries such as the aorta. The efficiency of PP is regulated by a degree of vascular stiffness (Safar et al., 2003) that influences vascular tone (Steppan et al., 2011), making it attractive as a blood pressure marker for this study.

A series of *multiple-group latent growth curve models* (MGLGCM), gauging both average trends across the population and differential growth rates of change across three domains—episodic memory (EM), visuospatial ability, and semantic memory—were specified to address the following research questions: (1) Do the allelic variants differ in rates of cognitive change? (2) Can pulse pressure (PP) elevation and older age magnify the influence of the Val^158^Met polymorphism on cognitive change? The confounding effects of years of education, sex, and cardiovascular diseases (CVDs), as well as chronological age and practice effects were accounted for. The MGLGCMs allow for evaluation of potential covariate interactions, enabling us to simultaneously rule out the potential confounding effects of an extensive set of characteristics that could potentially affect cognitive aging.

## Methods

### Participants

Written informed consent was obtained from all participants, and the study was approved by the regional ethics committee in Umeå, and performed in accordance with the Declaration of Helsinki. A sample of adults ranging from 35 to 85 years old at the first measurement occasion was drawn from the Betula study (Nilsson et al., 1997). The Betula study focused on memory, aging, and dementia in a sequential cohort, where new samples were added at each wave. The participants were randomly drawn from the Swedish population registry. Subjects were tested, interviewed, and medically examined on five occasions (1988–1990, 1993–1995, 1998–2000, 2003–2005, and 2008–2010; Nilsson et al., 1997; Persson et al., 2013a,b).

The baseline data collection for sample 1 started in 1988, baseline data for sample 3 was collected in 1993–1995, and participants in this study had repeat measurements on four occasions separated by 5 years. We recruited subjects from samples 1 and 3, since equivalent longitudinal data were available from these two samples. The original sample consisted of 1966 individuals.

We wanted to study the course of aging in healthy adults free from neurodegenerative diseases. Therefore, we excluded all participants who met the clinical criteria for dementia according to the Diagnostic and Statistical Manual of Mental Disorders (DSM IV, 1994) at the most recent data collection (time point 5; *n* = 278). An additional 103 persons were excluded due to missing genetic information (laboratory failure, non-compliance, or lost blood samples). The final sample consisted of 1585 subjects (Met/Met = 494, 31.2%; Val = 1091, 68.8%).

### Cognitive measures

The cognitive tasks were administered in the same way at all measurement occasions. Participants were individually tested during two test sessions, which lasted between 1.5 and 2 h for each participant. Five tasks of verbal episodic memory were used as manifest indicators in a multivariate latent growth curve model. Two individual tests were used to gauge semantic memory and spatial ability.

#### Episodic recall

The tasks included in the Betula study were rooted in extant theories of memory (Tulving, 2001), and were designed for the study but evaluated and tested in previous experiments, showing moderate to high reliability and stable coefficients (Nyberg et al., 2003). Structural equation models conducted on the Betula samples have shown good construct validity for episodic memory (Nyberg, 1994; Nyberg et al., 2003). Subjects were presented with four different wordlists, each including 12 nouns. The items were read aloud at a pace of two sec/item. Afterward, subjects repeated as many words as possible at a given pace (two sec/word), counted out by a metronome. For one list, the study/retrieval was performed under full attention. Study retrieval in the other lists was combined with performing a secondary task. This task consisted of sorting red and black cards into piles based on color. In one task, attention was divided during study but not during retrieval. In another task, the distraction was applied during retrieval but not during study. In the final task, distraction occurred both during study and retrieval. The order between the four conditions was balanced. Further, tasks of source recall were included where the participants were asked to recall whether sentences had been presented as subject-performed or verbal.

#### Visuospatial ability

The block design (BD) test from the Wechsler Adult Intelligence Scale (Wechsler, 1981) was used as an indicator of spatial ability. The participants were asked to place red and white blocks such that they were in line with a two-dimensional target pattern. The correct response was identical to the criterion figure, but three-dimensionally rotated in space. The maximum score was 51 points. The task was administered and scored according to standard instructions (Wechsler, 1981).

#### Semantic memory: vocabulary

SRB:1 (Dureman, 1960), a test of vocabulary (Vo), was treated as a proxy for semantic memory. SRB:1 is a multiple-choice test of word synonyms, containing 30 items with an administration time of 7 min. The test was scored according to SRB:1 criteria. For a full description of the cognitive tasks in the Betula study (see e.g., Nilsson et al., 1997; Persson et al., 2013a,b).

### Self-rated cardiovascular health and medication

A self-reported questionnaire about physicians' diagnoses was used to assess cardiovascular health. Responses were coded as “yes” or “no” with respect to the presence of hypertension, diabetes, and stroke. Further, self-reported information about the use of anti-hypertensive medication (ACE inhibitors, beta-blockers, alpha-blockers, diuretics, and calcium channel blockers) at baseline was included. Subjects were considered hypertensive in the presence of a physician's diagnosis and/or if they were currently using anti-hypertensive medication, in order to include participants with medically controlled hypertension. An index variable ranging from 0 to 3 was created. All the variables were inter-correlated and ranged from *r* = 0.080, *p* = 0.0001 to *r* = 0.158, *p* = 0.0001).

### Blood pressure

Resting blood pressure was obtained using auscultation after 5 min of rest in the supine position, and registered to the nearest five mmHg. Pulse pressure (PP), i.e., the difference between systolic and diastolic blood pressure, was then calculated.

### Genotyping of *COMT* Val^158^Met

Genomic DNA was isolated from whole blood with a Qiagen Genomic DNA Purification Kit (Qiagen, Chatsworth, CA, USA). Polymerase chain reactions were performed using HotstarTaq polymerase (Qiagen) in a total volume of 20 μL containing 1.5 mM MgCl_2_, 0.15 μM primers (fw: 50-TCA CCA TCG AGA TCA ACC CC-30, rev: 50-ACA ACG GGT CAG GCA TGC A-30), and ~50 ng of genomic DNA. After an initial 15 min denaturation step at 95°C, 45 cycles were performed including 30 s at 94°C, 30 s at 62°C, and 30 s at 72°C. PCR products were genotyped with a Pyrosequencer PSQ 96 and a PSQ 96 SNP Reagent Kit (Pyrosequencing, Uppsala, Sweden; Nordfors et al., 2002), using the sequencing primer 50-TGG TGG ATT TCG CTG-30.

## Statistical analyses

### Latent growth curve modeling

**Figure 1:**
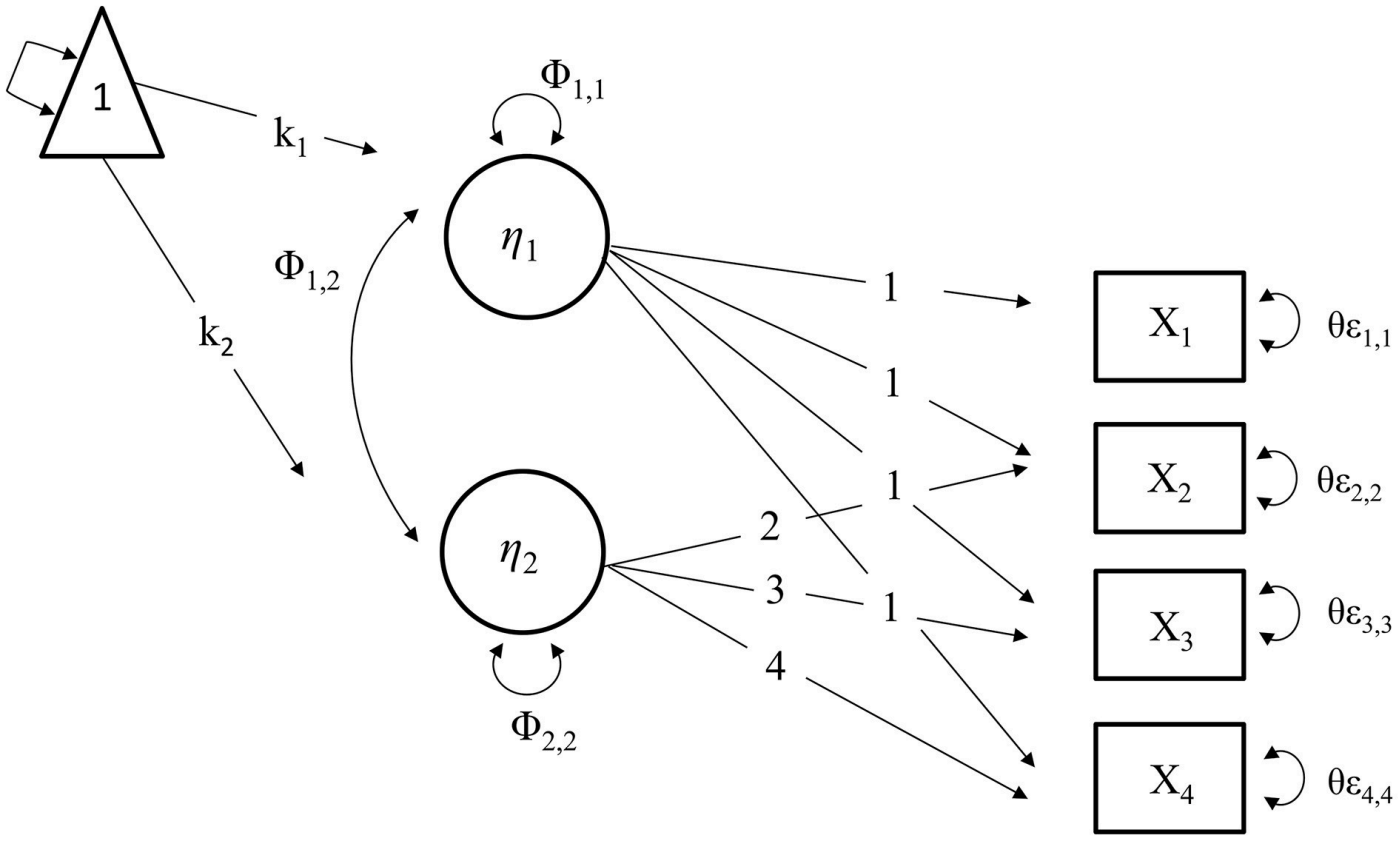
**The triangle indicates that the model includes means and intercepts**. The latent variable variables η_1_ represents the intercept and η_2_ the slope. The model has a mean slope (κ_2_), and an intercept (κ_1_). Φ_1, 1_Φ_2, 2_ denote variances in intercept and slopes, and Φ_1, 2_ covariance between the intercept and slope. X1-4 is the manifest indicators' repeated measures. In our application, either repeated measures of the Vocabulary and Block Design tasks are plugged in. θε_1, 1−4, 4_ represent the residuals of the measured variables. Intercepts of the manifest indicators are omitted from the path diagram.

**Figure 2:**
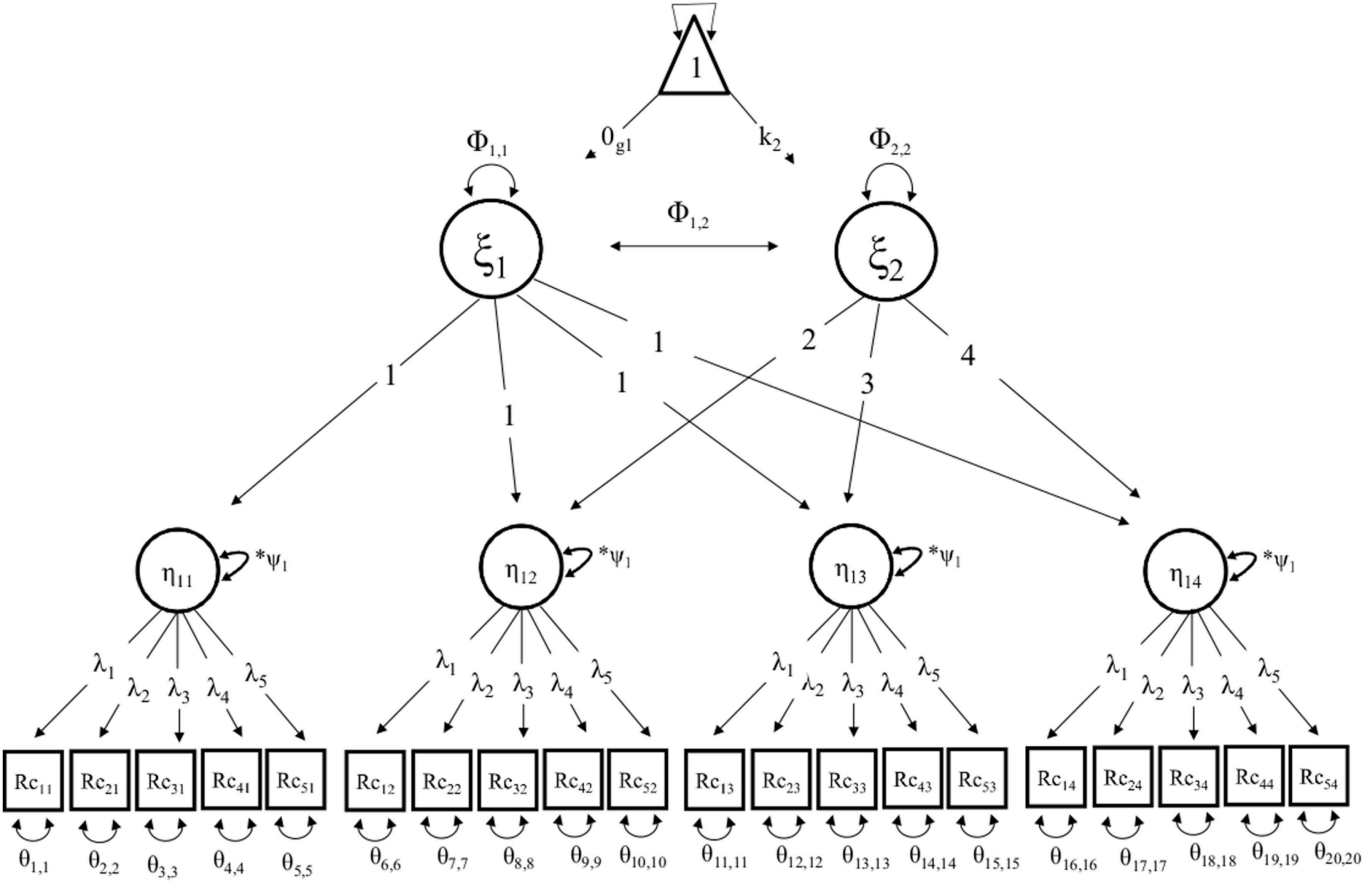
**A path diagram illustrate the second-order growth model, where η_1-4_ represents the latent construct Episodic Memory, over four time points, with four occasions of measurement, and five observed variables of verbal episodic memory at each measurement occasion**. The second order variables ξ_1_ represents the intercept and ξ_2_ the slope. The triangle indicates that the model includes means and intercepts. The model has a mean slope (κ_2_), and an intercept (κ_1_). ψ, represent the variance of the latent variable. The average intercept is constrained in group 1 for identification (0_*g*1_). Further do Φ_1, 1_Φ_2, 2_ denote variances in intercept and slopes, and Φ_1, 2_ covariance between the intercept and slope. θ_1, 1−20, 20_ are the residuals of the measured variables. Unique covariances and intercepts of the manifest indicators are omitted from the path diagram for parsimony.

The term *growth curve analysis* represents the process of describing, testing hypotheses, and making inferences about growth and change patterns of time-related phenomena (McArdle and Grimm, 2010). Individual differences in change are defined by latent variables (Muthén and Curran, 1997; Frank et al., 2007). Roughly speaking, such models capture both fixed effects describing average trends across the population, and latent or random effects that reflect a random probability distribution around that fixed effect (Curran et al., 2010). The simplest forms of *latent growth curve models* (LGCMs) are univariate with two latent variables: initial level (intercept) and growth rate over time (slope; see Figure 1; Curran and Muthén, 1999). The two latent variables are represented by a single indicator of repeated measures (see Figure 1). In cases of multiple indicators, a *second-order growth model* (see Figure 2) can be specified, where changes in the first-order latent variables (common factors) are carried by the second-order latent variables representing the intercept and slope (see Figure 2).

*Multiple-group latent growth curve models* (MGLGCMs) can simultaneously derive parameter estimates across groups and test the equivalence of measures. If an *unconditional growth model* is fitted to the pooled sample, the parameters that define the growth model are precisely equal across groups. When the grouping variable is added as a covariate, this will only introduce differences in the conditional means of the growth factors such that one of the groups will start higher or lower compared with the other and increase more or less steeply (DSM IV, 1994; Nyberg, 1994; Nyberg et al., 2003). Further, underlying differences in variances between groups may introduce bias into the parameter estimates. MGLGCM can address this issue (Curran et al., 2010; Curran and Muthén, 1999), as it performs simultaneous estimation of growth models across two or more groups. The presence of interactions can be examined in the context of MGLGCM by constraining and freeing parameter estimates across groups, and also by evaluating constraints by changes in model fit (Meredith, 1993; Gregorich, 2006; Persson et al., 2015).

### Model specification

A series of models were evaluated following five steps: (1) estimation of longitudinal factor models to establish equivalence of measures over time and across groups; (2) one-group analyses were performed on Met/Met and Val carriers, respectively, to evaluate the optimal fitting functional form within each group, and to unravel the presence of differential rates of change; (3) two-group analysis, including the Met/Met and Val groups simultaneously; (4) covariates were added to the models in the presence of significant variance; and (5) interaction was evaluated based on the fit of the parameter equality constraints.

To test if the same metric held over measurement occasions and across allelic groups, we first tested if there was substantial loss-of-model fit in comparing *longitudinal factor models* with free parameters to models with weak-to-strong factorial invariance (Meredith, 1993; Gregorich, 2006; McArdle, 2009).

*Unconditional growth models* were estimated to establish that the shape of growth curves was similar across the two groups. To determine the shape of the trajectory, a non-linear functional form was estimated by a latent basis model where a random slope was specified to be free from the second measurement interval, in addition to a linear model (Meredith and Tisak, 1990; Grimm and Ram, 2009a,b). The models were nested and compared by means of chi-square goodness-of-fit indices. In both models, the slopes were centered at the first time point (T1).

Growth factors were specified for intercept and slope, considering both mean and individual variation, and covariance between initial levels (intercepts) and slopes. The growth factors, residuals, and correlated uniquenesses were allowed to vary across groups. The conditional mean was constrained in the reference group (Met/Met carriers) for model identification. Univariate growth models were arranged for repeated measures of vocabulary (Vo) and block design (BD) scores (see Figure 1). A psychometric model was specified to represent the episodic memory (EM) with a second-order growth model, comprised of four occasions of repeated measures of five observed variables of verbal recall.

Differences in average cognitive change rates between the allelic variants were tested to evaluate equality of parameters across groups using the Wald Test of Parameter Constraints.

*Conditional growth models* were specified for the cognitive domains showing significant individual variance in cognitive trajectories. Based on the existing literature supporting a Val dominant model in relation to cognitive function (de Frias et al., 2004, 2005; Barnett et al., 2008; Wishart et al., 2011) and lower enzyme activity in Met carriers compared to Val carriers (Lachman et al., 1996), we divided the sample into two groups comprised of Met homozygotes and Val heterozygotes. The confounding effects of years of education, sex (women = 1), and cardiovascular diseases (CVDs) were controlled in the analyses. Further, pulse pressure (PP) and chronological age were grand-mean centered and used as continuous time-invariant covariates. To avoid underestimation of age-related changes, we estimated retest effects by a retest variable coded 0, 1, 2, or 3 (0 for baseline; Ferrer et al., 2005; Ghisletta et al., 2014). The presence of interaction was tested by evaluating equality constraints on the age slope using the difference in χ^2^ ($\Delta \chi^{2}=\chi_{\text{restricted}}^{2}-\chi_{\text{unrestricted}}^{2}$, with *df* = *df*_restricted_ − *df*_unrestricted_; Persson et al., 2015). A set of follow-up analyses was also performed on a subsample of participants free of CVDs (*n* = 1086).

Evaluation of the models fit to the data was done using conventional cut-off criteria from several fit indexes: the Comparative Fit Index (CFI) > 0.95, the Standardized Root Mean Square Residual (SRMR) < 0.08, and the Root-Mean-Square Error of Approximation (RMSEA) < 0.08 (Browne and Cudeck, 1993; Hu and Bentler, 1998, 1999), in addition to the χ^2^ test with its degrees of freedom (*df*). We assumed that data were missing at random, and parameter estimates were obtained using full information maximum likelihood (FIML) estimation (McArdle and Nesselroade, 1994; Little, 1995). In FIML, parameters are estimated based on all available data, and the key assumption is that the missing data are *missing at random* (MAR). Auxiliary variables related to the missingness mechanism were included in the analyses to further reduce estimation bias (Collins et al., 2001).

## Results

### Descriptive statistics and correlations

Descriptive statistics are presented in Supplementary Tables 1, 2. The genotype distribution of the *COMT* Val^158^Met polymorphism among the 1585 subjects conformed to Hardy-Weinberg equilibrium (χ^2^ = 0.022, *p* = 0.881), with 31.2% being Met/Met carriers, 19.7% being Val/Val carriers, and 49.1% being Val/Met carriers. Prior to the analyses, we identified potential outliers by Tukey upper and lower fences, multiplied by a factor of 2.2 [3Q (3rd quartile) + 2.2 × IQR (inter-quartile range)], and 1Q - 2.2 × IQR (Hu and Bentler, 1999). Ten univariate outliers in blood pressure were eliminated. The vocabulary (Vo) tasks exhibited negative skew (skewness from −1.248 to −1.454).

In homozygote Met carriers, the correlations of stability among variables ranged from *r* = 0.317 for source recall over T1-T4 to *r* = 0.507 over T1-T2 for full attention retrieval. The correlations for visuospatial ability, measured by a single task [block design (BD)], ranged from *r* = 0.736 over T1-T4 to *r* = 0.812 over T1-T2, and for Vo scores (a proxy for semantic memory) from *r* = 0.824 over T1-T4 to *r* = 0.855 over T1-T2 among the Met/Met carriers.

For Val heterozygotes, correlations between measurement occasions ranged from *r* = 0.237 for source recall over T1-T4 to *r* = 0.438 over T1-T2 for full attention retrieval. The correlations for BD ranged from *r* = 0.736 over T1-T4 to *r* = 0.813 over T1-T2, and for Vo scores from *r* = 0.856 over T1-T2 to *r* = 0.856 over T1-T2 in the Val group.

Logistic regressions were carried out to examine potential causes of attrition. Men (*B* = −0.318, *SE* = 0.139, *p* = 0.022) and older individuals (*B* = 0.041, *SE* = 0.007, *p* = 0.001) were more likely to drop out, after accounting for pulse pressure (PP; *p* = 0.375), cardiovascular diseases (CVDs; hypertension, stroke, and diabetes; *p* = 0.409), years of education (*p* = 0.275), and *COMT* allelic variation (*p* = 0.061).

### Longitudinal factor models: measurement invariance

To examine measurement invariance over time and across groups, *longitudinal factor models* were carried out in each group defined by allelic variant, followed by a multiple group analysis (2-group analysis). The factor loadings showed moderate to strong association with the latent construct episodic memory. The standardized factor loadings ranged from 0.466 to 0.755, in homozygote Met carriers, and from 0.444 to 0.745 in carriers of at least one Val allele. No substantial loss of fit was observed when comparing the baseline model with free parameters with the model assuming strong factorial invariance, showing good fit to the data in homozygote met carriers [$\chi_{\left(158\right)}^{2}=256.857$, CFI = 0.966, SRMR = 0.059, RMSEA = 0.036 (90% C.I. 0.027–0.043)] and Val carriers [$\chi_{\left(158\right)}^{2}$ = 402.733, CFI = 0.958, SRMR = 0.063, RMSEA = 0.038 (90% C.I. 0.033–0.042)]. Without crucial loss of fit compared to the free parameter models, the strong factorial multiple group model showed good fit to the data [$\chi_{\left(327\right)}^{2}$ = 733.983, CFI = 0.953, SRMR = 0.070, RMSEA = 0.040 (90% C.I. 0.036–0.043)], so that measurement invariance was established over the allelic groups.

### One-group analyses: functional form of cognitive trajectories and variance

As mentioned in Section Model Specification, we tested the functional form of trajectories by using linear or non-linear models. The trajectory shape was best described in a linear fashion across the allelic groups for EM and BD. The non-linear model fit closer to the Vo data (smaller χ^2^, RMSEA, and SRMR values; see Supplementary Table 3). The univariate LGCMs for Vo and BD showed good fit to the data [CFI = 1.000, 0.998, SRMR = 0.020, 0.037, RMSEA = 0.000, 0.038 (90% C.I. 0.000–0.030, 0.015–0.060), $\chi_{\left(10,10\right)}^{2}$ = 9.733, 21.351, *p* = 0.4642, 0.0188]. As presented in Table 1, variance in intercepts was present across all cognitive domains over allelic groups, but only EM scores exhibited significant slope variance across the two allelic groups. Consequently, determinants of individual rates of change could be added to the EM models, but their influence on the BD or Vo trajectories could not be evaluated.

**Table 1:** **Parameter estimates from the linear models, means of the initial level and growth rates, and their variances**.

	**Met/Met**			**Val**		
	**EM**	**BD**	**Vo**	**EM**	**BD**	**Vo**
k_1_	0.000 (0.000)	**2.705** (0.048)	**22.024** (0.244)	0.000 (0.000)	**2.676** (0.032)	**21.832** (0.157)
k_2_	**−0.138** (0.017)	**−0.118** (0.012)	0.015 (0.053)	**−0.123** (0.011)	**−0.116** (0.008)	−0.049 (0.038)
Φ_1, 1_	**1.124** (0.112)	**0.964** (0.073)	**25.886** (1.894)	**0.875** (0.059)	**0.922** (0.048)	**24.042** (1.169)
Φ_2, 2_	**0.017** (0.008)	0.004 (0.005)	0.016 (0.113)	**0.012** (0.005)	0.002 (0.004)	**0.304** (0.089)
Φ_1, 2_	−0.023 (0.023)	0.020 (0.015)	0.252 (0.343)	0.011 (0.012)	**0.040** (0.010)	0.159 (0.249)

*EM, Episodic Memory; BD, Block Design; Vo, Vocabulary; k_1_, average intercept; k_2_, average slope; Φ_1, 1_, intercept variance; Φ_2, 2_, slope variance; Φ_1, 2_, intercept and slope covariance; Standard errors are presented in parentheses*.

*Bold faced parameter estimates indicate significance (p < 0.05)*.

### Multiple-group latent growth curve models: two-group analyses

The chi-square distribution for the EM model for Met/Met carriers was 320.273, and 492.008 for Val heterozygotes. The standardized factor loadings were moderate to strong, ranging from 0.495 to 0.730. As presented in Table 1, the parameter estimates of average growth rates were very similar in size across groups, which was confirmed by the insignificant differences between the parameters {*p* = n.s. [e.g., EM: $\Delta \chi_{\left(1\right)}^{2}=0.407$, *p* = 0.5235 (Wald Test of Parameter Constraints)]}.

### Determinants of differential rates of change

As mentioned previously, covariates were added selectively to the EM model since variance in change was present in both allelic groups. All results are presented in Table 2. When the sample was analyzed in its entirety, we observed no magnifying effects of age on the association between *COMT* and EM decline [$\Delta \chi_{\left(1\right)}^{2}$ = 1.164, *p* = n.s.]. However, we observed additive effects of age on the EM slope, so that older Val carriers showed increased decline, accounting for the demographic factors, after (β = −0.433, *SE* = 0.170, *p* = 0.010), exclusion of the four younger cohorts (35, 40, 45, and 50 years) [$\Delta \chi_{\left(1\right)}^{2}$ = 128,327 *p* = 0.0001]. The association was attenuated after additional control for cardiovascular risk factors (*p* = 0.130). No influence of retest effects on the EM trajectories emerged. Women showed higher baseline EM scores than men in both allelic variants, but sex did not significantly differentiate growth rates in any allelic group. Higher educational attainment influenced intercept level, but had no effect on individual differences in change.

**Table 2:** **Covariates effects on initial level and change: Raw and standardized parameter estimates**.

	**Met/Met**				**Val**			
	**Level**		**Slope**		**Level**		**Slope**	
	**Unst**.	**Std**.	**Unst**.	**Std**.	**Unst**.	**Std**.	**Unst**.	**Std**.
Age	−0.028 (0.004)^*^	−0.374 (0.047)^*^	−0.075 (0.021)^*^	−0.361 (0.097)^*^	−0.024 (0.003)^*^	−0.365 (0.038)^*^	−0.047 (0.023)^*^	−0.272 (0.135)^*^
Sex	0.465 (0.079)^*^	0.222 (0.037)^*^	−0.905 (0.584)^*^	−0.156 (0.102)	0.300 (0.047)^*^	0.161 (0.025)^*^	−0.185 (0.381)	−0.038 (0.078)
Education	0.048 (0.009)^*^	0.222 (0.041)^*^	0.115 (0.078)	0.194 (0.132)	0.082 (0.007)^*^	0.358 (0.030)	0.045 (0.056)	0.075 (0.095)
Practice	0.249 (0.038)^*^	0.268 (0.040)^*^	0.463 (0.380)	0.180 (0.140)	0.162 (0.026)^*^	0.187 (0.029)^*^	0.393 (0.376)	0.174 (0.160)
CVD	−0.137 (0.075)	−0.075 (0.041)	−0.065 (0.606)	−0.013 (0.119)	−0.168 (0.047)^*^	−0.100 (0.028)^*^	0.482 (0.429)	0.109 (0.099)
PP	−0.003 (0.003)	−0.047 (0.045)	0.013 (0.021)	0.077 (0.127)	0.004 (0.002)^*^	0.081 (0.032)^*^	−0.050 (0.015)^*^	−0.351 (0.115)^*^

*CVD, Cardio vascular disease; PP, Pulse pressure; Sex (1 = female, 0 = Male), Practice (0–3); Age; Chronological age centered on the sample grand mean; Unst., Non-standardized parameter estimates; Std, Standardized parameter estimates; STDYX solution. Standard errors of the parameter estimates are presented within parenthesis*.

* *Bonferroni-adjusted significance α′ = 0.01*.

**Figure 3:**
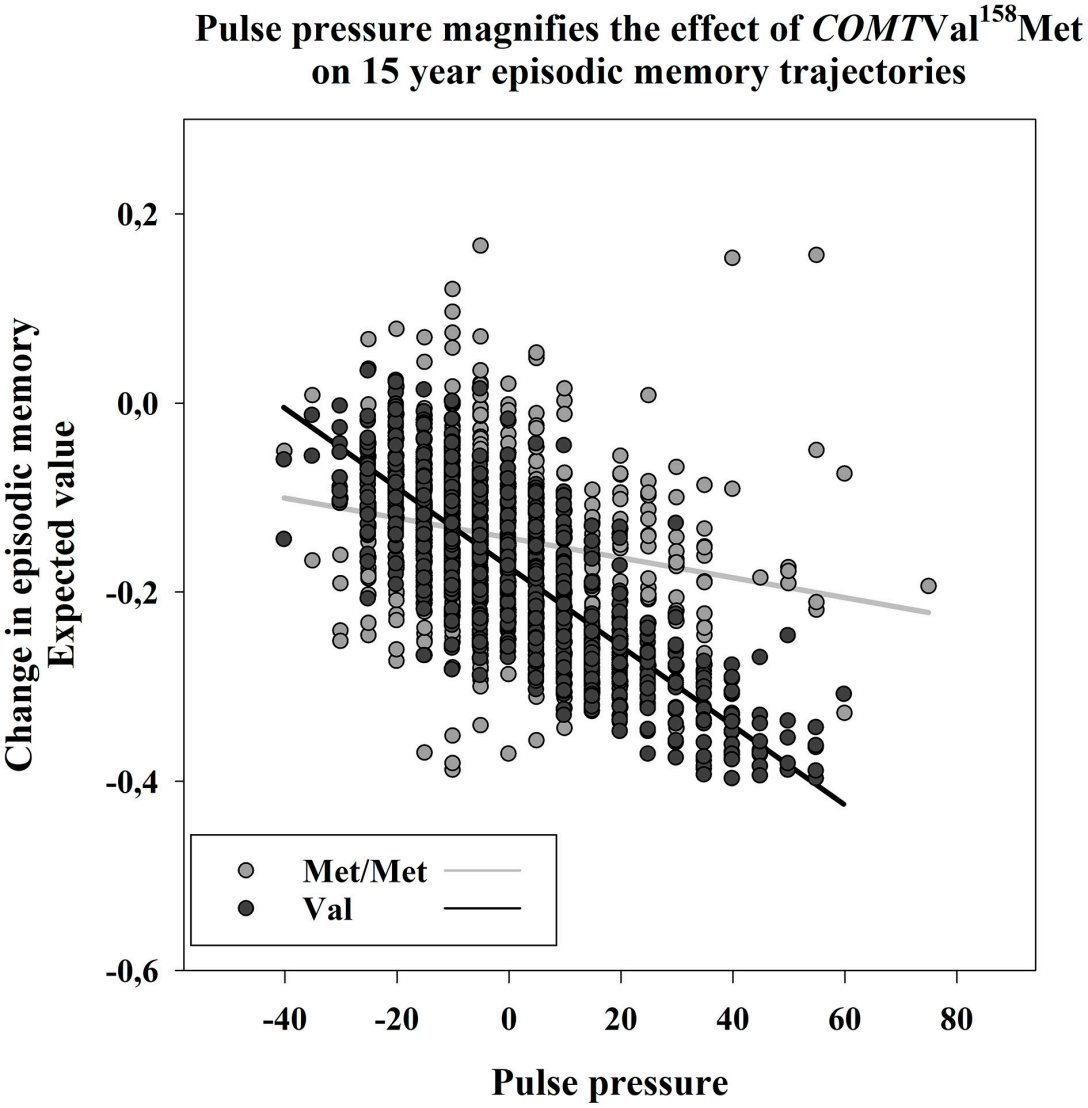
**Episodic Memory (EM) changes as a function of pulse pressure (PP) at baseline, in Met/Met, and Val carriers respectively**. PP magnifies the effect of *COMT* ^158^Val on 15 years EM change, resulting in greater decline in Val carriers. The expected values are calculated from the slope factor scores, while taking into account the effects of covariates. PP in millimeter of mercury is centered at the grand-mean.

The presence of CVDs at baseline was reflected in lower baseline EM scores in Val carriers, and a trend in the same direction was present in the Met/Met group (*p* = 0.068). There was no indication of magnifying effects of CVDs on the relationship between SNP and EM [$\Delta \chi_{\left(1\right)}^{2}=0.285$, *p* = n.s.]. Pulse pressure did not influence the level of EM performance across allelic groups (e.g., Val: *p* = 0.020, α′ = 0.010]. Val carriers declined in episodic memory as a function of PP elevation over time (*p* = 0.002, α′ = 0.01). The interaction effect was present by means of significant deterioration of fit by the parameter equality constraint [$\Delta \chi_{\left(1\right)}^{2}$ = 5.773, *p* = 0.005]. See Figure 3 for an illustration.

### Subsidiary analyses: subjects without CVDs

The analyses were carried out after excluding individuals with CVDs (stroke, diabetes, or hypertension; Val *n* = 751, Met/Met *n* = 335). It is worth noting that individuals without CVDs were younger than persons suffering from CVDs (*F*_(3)_ = 110.041, *p* = 0.0001]. Higher levels of PP still indicated EM decline among *COMT* Val carriers (β = −0.437, *SE* = 0.191, *p* = 0.022). This interaction was confirmed by significant deterioration of model fit [$\Delta \chi_{\left(1\right)}^{2}$ = 5.773, *p* = 0.005]. Incremental practice effects on episodic memory growth (β = 0.354, *SE* = 0.140, *p* = 0.015) were selectively evident in Met/Met carriers, but the slope constraint was not tenable [$\Delta \chi_{\left(1\right)}^{2}$ = 2.30, *p* = 0.12].

## Discussion

Our chief finding was that pulse pressure (PP) elevation moderated the effect of the functional Val^158^Met polymorphism on 15-years episodic memory (EM) trajectories, leading to steep decline in Val carriers and accounting for various covariates with the potential to affect individual differences in cognitive change. The effect was still present when individuals with a history of hypertension, diabetes, or stroke were excluded. To the best of our knowledge, this is a novel finding. We further confirmed previous reports showing that aging magnified the influence of genetic variance in a common functional genetic polymorphism, Val^158^Met, leading to greater decline of EM scores in Val carriers, at least when potential attenuation from demographic factors was taken into account. The effects were not moderated by any other covariates.

Importantly, we did not detect any evidence of differences between allelic groups in rates of change across cognitive markers. Instead, we found that both groups conformed to frequent reports of stability rather than change in crystalized abilities (such as vocabulary) and average decline in fluid abilities during aging (Horn and Cattell, 1967; Rönnlund et al., 2005; Ghisletta et al., 2012).

Individual single nucleotide polymorphisms (SNPs) may have little influence on complex behavioral measures such as cognitive function, as even whole genes may exert small effects (Plomin et al., 1994). Our results conform to findings from a large meta-analysis that reported no association between Val^158^Met and a wide range of cognitive functions (Barnett et al., 2008). The negative findings reported herein also contradict previous reports of allele-specific differences in EM and spatial performance scores (de Frias et al., 2004, 2005). It is important to note that these authors applied a single measurement interval (5 years) to a male sample. It is possible that our findings would have been similar if these design aspects were equivalent. Variability in task characteristics between studies may further enhance differences in results.

Advanced age predicted lower EM scores at inception, followed by decay over 15 years in both allelic variants after controlling for the potential confounding of practice effects. There was no indication of additive effects of age when the sample was analyzed in its entirety. Because previous work has illustrated that the effects of *COMT* may emerge in older adults and individuals at risk for cognitive decline (de Frias et al., 2005; Lindenberger, 2008; Nagel et al., 2008; Josefsson et al., 2012; Papenberg et al., 2014), we performed a secondary analysis, excluding the younger age cohorts while controlling for demographic factors and retest effects. We indeed found support for the additive effects of age in the older Val allelic group, in accordance with the resource modulation hypothesis. This hypothesis proposes that the effects of genetic polymorphisms are magnified in older age following the reduction of brain resources that influence cognitive functions (Lindenberger, 2008). The effect, however, was attenuated by statistical control for cardiovascular diseases and pulse pressure. We would like to stress the importance of further assessing the influence of cardiovascular risk factors in age-related behavioral genetic studies in the future.

The effects on memory were not moderated by any other covariates. Sex did not magnify the influence of genetic risk on episodic memory trajectories, conforming to previous negative findings that considered the additive effects of SNPs and sex on cognition (Ghisletta et al., 2014). Higher educational attainment resulted in better initial EM scores across the allelic variants, but could not reliably explain differential EM changes. The combined influence of genetics and educational factors on cognitive function may gain ground in the context of heritability, likely reflecting polygenetic influences (Rowe et al., 1999) to a greater degree than in the candidate approach of a single SNP used in this study.

Practice gains were present on intercept levels of EM scores, but no additional time-related practice gains were observed across the two allelic variants after demographics and health-related effects were taken into account. Studies have previously reported diminishing practice gains on global cognitive function beyond follow-up (Jacqmin-Gadda et al., 1997). Gains in EM scores were present in the Met carriers after excluding individuals with diabetes, stroke, and hypertension. An interaction was not established by means of tenability of the equality constraint. This result deserves some attention from future studies, since the trend gives a hint of possible genomic variability differentiating practice gains by potentially influencing neurocognitive reserve (Satz, 1993) in carriers of lower enzyme activity alleles.

No influence of cardiovascular disease load was observed beyond the initial level. Greater cardiovascular disease load indicated lower initial EM scores among heterozygous Val carriers, and a trend in this direction was also observed in Met carriers (*p* = 0.068). One reason for the discrepancy in results between cross-sectional negative impacts and absence of long-term effects could be that participants were informed about their health status at baseline for ethical reasons, which could have reversed long-term cognitive decline due to lifestyle changes.

The Val allele is associated with higher *COMT* enzyme availability and has previously been associated with cognitive decline, although findings were mixed (Barnett et al., 2008; Wang et al., 2013). Our report shows that the negative influence of the Val allele is particularly pronounced with pulse pressure elevation. Importantly, this effect was present even after excluding individuals with diabetes, stroke, and hypertension, suggesting that the effect was also crucial in healthier subjects who would be expected to take advantage of greater cognitive reserve by maintaining their cognitive abilities (Stern, 2003).

The underlying biomedical mechanism of these effects is unknown. The mechanism behind the reported interaction may emerge from various pathways. A common explanation is that Val^158^Met influences cognitive function through the regulation of dopamine levels, with Val carriers having high COMT activity and lower dopamine levels (Lachman et al., 1996; Chen et al., 2004). The Val allele has also been linked with high blood pressure (Hagen et al., 2007; Kamide et al., 2007), which can be mediated by dopamine through its influence on sodium regulation (Jose et al., 2003; Zeng et al., 2007). Inflammation may be another variable accounting for this effect. Inflammation influences permeability in the vasculature (Kolattukudy and Niu, 2012) that can cause decreased blood flow to the brain, leading to increased white matter burden (Novak et al., 2006; Kolattukudy and Niu, 2012) that manifests as cognitive decline (Birdsill et al., 2013). Taken together, these factors may offset episodic memory decline.

The prodromal stage of Alzheimer's disease is known to be extensive (Small et al., 2003; Iacono et al., 2009), and even if demented individuals were excluded until the last available data collection, occasionally there may be a risk of including individuals with early dementia.

We used pulse pressure (PP), combining information from systolic and diastolic blood pressure, in the current report. Combining PP with the carotid-femoral pulse wave velocity (PWV) may have given a more precise measure of vessel tone and vascular stiffness. Our findings would benefit from future replication by combining such measures.

Polygenetic influences may shape episodic memory in aging, directly and indirectly, by affecting mediators of cognitive decline. Various genes have been proposed to influence hypertension (Friese et al., 2011), and SNPs that affect immune system pathways may influence verbal declarative memory (Debette et al., 2015). Such SNPs may enhance the effects of inflammation on aging and interact with the effects reported herein, but unfortunately, we lacked such information. Our findings should be replicated to enhance the generalizability of the results, and further investigation of biomedical mechanisms behind the effects is warranted.

To the best of our knowledge, this is the first report to show that pulse pressure magnifies the effects of the *COMT* Val allelic variant on 15-years episodic memory decline. This report underscores the importance of addressing synergistic effects on normal cognitive aging, as the addition thereof may also place healthy individuals at greater risk for memory decline.

## Author contributions

NP: research questions, statistical analyses and interpretation of data, drafting the manuscript, editing, and revising the manuscript; HF, CL, and AS: editing and critical revision.

### Conflict of interest statement

The authors declare that the research was conducted in the absence of any commercial or financial relationships that could be construed as a potential conflict of interest.
