# Supplementary material for: Pulse Pressure Magnifies the Effect of COMT Val158Met on 15 Years Episodic Memory Trajectories
Source: Front Aging Neurosci. 2016 Mar 2;8:34. doi: 10.3389/fnagi.2016.00034 (PMC4773588; doi:10.3389/fnagi.2016.00034)
Supplement: Supplementary file 1 [file Table1.docx]

Supplementary Table 1. Descriptive Statistics of Demographic and Cardiovascular Variables.

|  | Met/Met (*n=*494) | | Val (*n=* 1091) | |
| --- | --- | --- | --- | --- |
|  | Mean | Sd | Mean | Sd |
| Age (Years) | 56.81 | 23.816 | 57.19 | 14.104 |
| Education | 10.326 | 4.822 | 10.355 | 4.056 |
| Pulse pressure | 55.086 | 17.750 | 55.312 | 17.216 |
| Women (%) | 57.1 |  | 52.7 |  |
| CVD (%) | 31.6 |  | 30.9 |  |

*Note.* CVD = Cardiovascular disease; Pulse pressure in mmHg (millimeter of mercury).

Supplementary Table 2. Descriptive Statistics of the Dependent Variables.

|  | Met/Met (*n =* 494) | | | Val (*n =* 1091) | | |
| --- | --- | --- | --- | --- | --- | --- |
|  | Mean | Sd | *n* | Mean | Sd | *n* |
| FA_T1_ | 5.463 | 1.697 | 492 | 9.425 | 4.671 | 1083 |
| Drc_T1_ | 3.915 | 1.572 | 492 | 4.007 | 1.384 | 1078 |
| Drt_T1_ | 4.648 | 1.735 | 492 | 4.655 | 1.561 | 1079 |
| Drcrt_T1_ | 3.738 | 1.508 | 492 | 3.672 | 1.439 | 1077 |
| SoRc_T1_ | 9.779 | 3.127 | 494 | 10.151 | 4.400 | 1088 |
| FA_T2_ | 5.453 | 1.608 | 417 | 5.359 | 1.677 | 957 |
| Drc_T2_ | 4.053 | 1.488 | 414 | 4.006 | 1.399 | 944 |
| Drt_T2_ | 4.603 | 1.556 | 413 | 4.609 | 1.560 | 946 |
| Drcrt_T2_ | 3.763 | 1.408 | 414 | 3.747 | 1.412 | 945 |
| SoRc_T2_ | 10.458 | 3.233 | 415 | 14.333 | 3.379 | 958 |
| FA_T3_ | 5.383 | 1.661 | 350 | 5.274 | 1.731 | 758 |
| Drc_T3_ | 3.927 | 1.367 | 331 | 3.968 | 1.414 | 713 |
| Drt_T3_ | 4.542 | 1.366 | 332 | 4.646 | 1.540 | 711 |
| Drcrt_T3_ | 3.735 | 1.375 | 336 | 3.723 | 1.354 | 714 |
| SoRc_T3_ | 11.198 | 3.996 | 349 | 11.442 | 3.785 | 739 |
| FA_T4_ | 5.277 | 1.706 | 278 | 5.348 | 1.675 | 584 |
| Drc_T4_ | 4.574 | 1.651 | 249 | 3.853 | 1.365 | 539 |
| Drt_T4_ | 3.812 | 1.454 | 255 | 4.565 | 1.456 | 536 |
| Drcrt_T4_ | 3.680 | 1.294 | 250 | 3.639 | 1.360 | 540 |
| SoRc_T4_ | 11.408 | 3.705 | 267 | 13.015 | 2.001 | 573 |
| BD_T1_ | 26.982 | 10.717 | 494 | 26.679 | 5.239 | 1088 |
| BD_T2_ | 25.914 | 10.716 | 413 | 25.660 | 5.050 | 961 |
| BD_T3_ | 24.797 | 10.378 | 351 | 24.516 | 4.926 | 747 |
| BD_T4_ | 23.416 | 9.513 | 264 | 23.232 | 4.532 | 574 |
| Vo_T1_ | 21.975 | 5.503 | 494 | 21.752 | 5.239 | 1091 |
| Vo_T2_ | 22.381 | 5.416 | 427 | 22.278 | 5.050 | 970 |
| Vo_T3_ | 23.16 | 4.759 | 359 | 22.70 | 4.926 | 788 |
| Vo_T4_ | 23.07 | 4.922 | 273 | 23.04 | 4.532 | 599 |

*Note.* FA = Full attention at recall; Drc = distraction at recall; Drt = distraction at retrieval; Drcrt = distraction at recall and retrieval; SoRc = source recall; BD = Block Design; Vo = Vocabulary; T_1_,.., T_4_ = time points 1to 4.

Supplementary Table 3. Fit Statistics for the Unconditional Latent Growth Curve Models (LGCMs).

|  | Met/Met (*n=*494) | | Val (*n=*1091) | |
| --- | --- | --- | --- | --- |
| *EM* | Linear LGCM | Latent basis LGCM | Linear LGCM | Latent basis LGCM |
| Parameters | 64 | 66 | 64 | 66 |
| *χ^2^*(*df*) | 267.208 (166) | 265.092 (164) | 424.388 (166) | 411.210 (164) |
| RMSEA | 0.035 | 0.035 | 0.038 | 0.037 |
| 90% C.I. | 0.027- 0.043 | 0.027- 0.043 | 0.033- 0.042 | 0.033- 0.042 |
| SRMR | 0.060 | 0.060 | 0.064 | 0.065 |
| CFI | 0.965 | 0.965 | 0.955 | 0.957 |
| *BD* | Linear LGCM | Latent basis LGCM | Linear LGCM | Latent basis LGCM |
| Parameters | 9 | 11 | 9 | 11 |
| *χ^2^*(*df*) | 2.444 (5) | 1.693 (3) | 7.289 (5) | 6.918 (3) |
| RMSEA | 0.000 | 0.000 | 0.021 | 0.035 |
| 90% C.I. | 0.000 -0.041 | 0.000 - 0.061 | 0.000- 0.050 | 0.000- 0.069 |
| SRMR | 0.014 | 0.020 | 0.022 | 0.024 |
| CFI | 1.000 | 1.000 | 0.999 | 0.999 |
| *Vo* | Linear LGCM | Latent basis LGCM | Linear LGCM | Latent basis LGCM |
| *χ^2^*(*df*) | 9.746 (5) | 3.994 (3) | 11.605 (5) | 10.158 (3) |
| Parameters | 9 | 11 | 9 | 11 |
| RMSEA | 0.044 | 0.026 | 0.035 | 0.047 |
| 90% C.I. | 0.000-0.085 | 0.000 -0.084 | 0.007 -0.061 | 0.017 -0.080 |
| SRMR | 0.050 | 0.016 | 0.030 | 0.007 |
| CFI | 0.997 | 0.999 | 0.998 | 0.998 |

*Note.* EM = Episodic Memory; BD = Block Design; Vo= Vocabulary; *df* = degrees of freedom; C.I. = Confidence Interval; RMSEA = Root-Mean-Square Error of Approximation, SRMR = Standardized Root Mean Square Residual, CFI = Comparative Fit Index.
